# Patient Satisfaction With a One‐Stop Service Center in a Tertiary Hospital: A Cross‐Sectional Study

**DOI:** 10.1002/hsr2.73330

**Published:** 2026-10-18

**Authors:** Gong Xue, Chen Xia, Wei Li Li, Ding Xiao Rong, Peng Wei Qun

**Affiliations:** ^1^ Outpatient Department Xinhua Hospital Shenzhen Guangdong Province China; ^2^ Department of Nursing Peking University Shenzhen Hospital Shenzhen Guangdong Province China

**Keywords:** cross‐sectional study, one‐stop service, outpatient management, patient satisfaction, quality of care

## Abstract

**Objective:**

To investigate the current status of outpatient satisfaction and its key influencing factors under the “One‐Stop” service model, providing an empirical basis for optimizing outpatient service processes and enhancing operational efficiency.

**Methods:**

A cross‐sectional survey was conducted from March to April 2025, selecting 1016 outpatients (or their family members) from 15 departments in our hospital. The Patient Satisfaction Questionnaire (PSQ) was used for investigation, covering basic patient information, appointment experience, quality of doctor–patient communication, and overall satisfaction. One‐stop service convenience (11 items) and communication quality (4 items) were extracted as composite scores. Data were analyzed using SPSS version 27.0, employing descriptive statistics, Pearson's correlation, and hierarchical linear regression to identify independent predictors of satisfaction.

**Results:**

The overall satisfaction level among outpatients was relatively high, at 89.17% (906/1016). Pearson's correlation analysis revealed that both one‐stop convenience (*r* = 0.992, *p* < 0.001) and communication quality (*r* = 0.934, *p* < 0.001) were strongly associated with overall satisfaction. Hierarchical regression further identified both as significant independent predictors: one‐stop convenience (*β* = 0.767, *p* < 0.01) and communication quality (*β* = 0.254, *p* < 0.01), together explaining 99.9% of the variance in satisfaction (*R* ^2^ = 0.999). In contrast, demographic variables (age, gender) and binary waiting time (on‐time vs. delayed) were not significant in the multivariable model (all *p* > 0.05). Among the respondents, 94.69% (962/1016) expressed high recognition of the environmental convenience, and within this group, the excellent satisfaction rate reached 96.67%.

**Conclusion:**

The “One‐Stop” service model can effectively enhance outpatient satisfaction. Service process efficiency (one‐stop convenience) is the dominant driver of satisfaction, while the quality of doctor–patient communication serves as a distinct and complementary contributor. Environmental convenience remains a foundational element. These three factors should be key focuses in the continuous improvement of healthcare quality.

## 1 Introduction

Patient satisfaction is a pivotal metric for evaluating healthcare quality, serving as a direct reflection of a hospital's clinical and nursing standards while also exerting a profound impact on treatment adherence and health outcomes [1]. Within outpatient settings of large public hospitals, patient satisfaction embodies a composite measure influenced by the quality of doctor–patient communication, service efficiency, and environmental amenities, thereby constituting a fundamental basis for operational and managerial optimization. Supporting the value of integrated care models, King and colleagues documented a decade of follow‐up data from a New Zealand regional hospital utilizing a one‐stop clinic for dermatological management, demonstrating its capacity to deliver efficient, streamlined, and cost‐effective community‐based care. Notably, the model's effectiveness persisted following its implementation in additional regional centers [2].

Amidst globally rising life expectancy and consequent expansions in health service provision, public hospitals in China—as the cornerstone of the healthcare system—shoulder substantial patient loads while navigating the dual challenges of ongoing medical reform and the imperative to enhance patient experience [3]. In this context, the establishment of One‐Stop Service Centers in outpatient departments has emerged as a crucial strategy to elevate service quality and efficiency. Through resource integration and process re‐engineering, these centers significantly reduce waiting times and improve the overall patient journey [3, 4, 5, 6]. Theoretically, the SERVQUAL model [7] provides a useful lens for understanding these improvements, positing that service quality is determined by the gap between customer expectations and perceptions across five dimensions: reliability, responsiveness, assurance, empathy, and tangibles. The One‐Stop model, by streamlining processes and centralizing services, has the potential to narrow this gap across multiple dimensions simultaneously.

Nevertheless, while existing research has largely concentrated on Western healthcare systems, a significant gap remains in empirical evidence regarding the One‐Stop Service model within the unique Chinese public hospital context—characterized by high patient volume, distinct sociocultural factors, and reform pressures. Moreover, prior studies have often examined service efficiency (e.g., waiting time) and interpersonal quality (e.g., communication) as separate predictors of satisfaction [1, 4, 8], without simultaneously quantifying their relative importance within a unified service model. It remains unclear whether process efficiency or humanistic care is the dominant driver of satisfaction in a high‐volume, real‐world One‐Stop setting.

Consequently, this study employs a large‐sample, cross‐sectional survey of 1016 patients to quantitatively analyze the impact of the outpatient One‐Stop Service model on patient satisfaction in a Chinese public hospital. Specifically, we aimed to (1) assess the overall level of patient satisfaction under the One‐Stop model; (2) identify the independent contributions of one‐stop service convenience and communication quality to satisfaction using hierarchical regression; and (3) determine whether demographic factors and waiting time remain significant predictors after accounting for these service‐quality dimensions. The findings aim to provide localized, evidence‐based insights to inform the optimization of outpatient services tailored to the Chinese healthcare landscape.

## 2 Materials and Methods

### 2.1 Study Design and Participants

This cross‐sectional study was conducted at Xinhua Hospital, a tertiary public general hospital in Shenzhen, Guangdong Province, China. The hospital serves more than 500 outpatients per day across 22 clinical departments, including Orthopedics, General Surgery, Gynecology, Otolaryngology, Respiratory Medicine, Cardiovascular Medicine, and Endocrinology.

### 2.2 Participants and Sampling

A convenience sampling method was used to recruit nonemergency outpatients from 15 departments in our hospital between March and April 2025. A total of 1020 questionnaires were distributed, and 1016 valid questionnaires were collected, yielding an effective response rate of 99.6%. The inclusion criteria were as follows: (1) outpatients aged ≥ 18 years; (2) clear consciousness and basic literacy skills; and (3) if patients were unable to complete the questionnaire due to age, cognitive, or physical reasons, their primary accompanying family members could assist in filling it out. Exclusion criteria included patients with severe acute illnesses or cognitive impairment that prevented informed consent.

### 2.3 Sample Size

The sample size was informed by both precision‐based estimation and the events‐per‐variable rule for multivariable regression. Using a pilot test (*n* = 30) in which the Overall Satisfaction Score (OSS, range 15–75) showed a standard deviation (SD) of approximately 3.7, and allowing a margin of error of 1.5 points at 95% confidence (*z* = 1.96), the required sample size was *n* = (1.96^2^ × 3.7^2^)/1.5^2^ ≈ 23. To accommodate up to 10 predictors in hierarchical regression (rule of 10 observations per parameter), a minimum of 100 participants was desirable. Anticipating a high response rate under convenience sampling, we distributed 1020 questionnaires and obtained 1016 valid responses (99.6%), far exceeding the minimum requirement. Post hoc precision check: with *N* = 1016 and SD = 3.70, the 95% CI half‐width for the mean OSS was 1.96 × 3.70$/\sqrt{1016}$ ≈ 0.23 points, well within acceptable bounds for the 15–75 scale.

### 2.4 Research Instruments

Data were collected using a self‐administered questionnaire specifically designed for this study. The questionnaire comprised two sections. The first section captured general patient information, including demographics (age, gender), appointment mode, and waiting experience (whether the appointment was on schedule or delayed). The second section was a self‐designed 15‐item Patient Satisfaction Scale developed by the research team based on the SERVQUAL framework [7] and the operational characteristics of the hospital's One‐Stop Service Center. Each item was rated on a 5‐point Likert scale (1 = *very dissatisfied* to 5 = *very satisfied*). The scale covered four domains: one‐stop service convenience (11 items: registration flow, waiting time at service windows, pharmacy and examination waiting, guidance and signage, physical environment, cleanliness, and complaint‐handling convenience); communication quality (4 items: physician attitude, nurse attitude, physician technical competence, and nurse technical competence); and overall satisfaction (1 item, scored as part of the total). The sum of all 15 items formed the OSS (range 15–75).

### 2.5 Validity and Reliability

Content validity was assessed by a panel of three senior clinicians and two health service researchers; minor wording adjustments were made based on their feedback. A pilot test among 30 outpatients yielded a Cronbach's *α* of 0.91 for the full scale, indicating satisfactory internal consistency.

### 2.6 Data Collection Procedure

Trained research assistants distributed the self‐administered questionnaires to eligible participants immediately after their outpatient consultation. Participants completed the questionnaire in a designated quiet area, taking approximately 10–15 min. Completed questionnaires were collected on‐site. Questionnaires with more than one missing item per domain were excluded from analysis (*n* = 4, 0.4% of returned questionnaires). For the remaining questionnaires, missing items (less than 1% of all responses) were imputed using the mean of the nonmissing items within the same domain.

### 2.7 Statistical Analysis

Data were analyzed using SPSSAU (Version 25.0; SPSSAU Project, Guangzhou, China; https://www.spssau.com) [9]. Descriptive statistics were used to summarize participant characteristics: continuous variables were presented as mean ± SD, and categorical variables as frequencies (*n*) and percentages (%).

To examine the relationships between service‐quality dimensions and overall satisfaction, the following analyses were performed:

(1) Pearson's correlation analysis was conducted to assess the bivariate associations between the composite scores (one‐stop convenience mean, communication quality mean) and the OSS.
(2) Independent‐samples *t*‐test and one‐way analysis of variance (ANOVA) were used to compare OSS across demographic subgroups (gender, age group, waiting time status).
(3) Hierarchical linear regression was employed to identify the independent predictors of overall satisfaction. Variables were entered in three blocks: Model 1 included demographic characteristics (gender, age group) and waiting time status (on‐time vs. delayed); Model 2 added the one‐stop convenience score; Model 3 added the communication quality score. Standardized beta coefficients (*β*), unadjusted *B* coefficients with 95% confidence intervals (CI), *R* ^2^, change in *R* ^2^ (Δ*R* ^2^), and variance inflation factor (VIF) were reported.

All tests were two‐tailed, and a *p* value of less than 0.05 was considered statistically significant.

### 2.8 Ethical Considerations

This cross‐sectional survey, which utilized anonymous questionnaires, qualified for exemption from informed consent. The study relied exclusively on secondary data from publicly available, de‐identified source. We confirm that all procedures were conducted in accordance with the institutional ethical standards and the principles for medical research involving human subjects as outlined in the Declaration of Helsinki.

### 2.9 Reporting Guideline

This study was reported in accordance with the STROBE (Strengthening the Reporting of Observational Studies in Epidemiology) statement for cross‐sectional studies [10]. The completed STROBE checklist is provided as a Supporting File S1.

## 3 Results

### 3.1 Demographic and Clinical Characteristics

A total of 1016 participants completed the survey. The sample consisted of 611 females (60.14%) and 405 males (39.86%). Participants were distributed across 4 age groups: ≤ 30 years (*n* = 320, 31.50%), 31–40 years (*n* = 290, 28.54%), 41–50 years (*n* = 188, 18.50%), and ≥ 51 years (*n* = 218, 21.46%). Regarding appointment waiting time, 825 participants (81.2%) were seen on time, while 191 (18.8%) experienced delays. The OSS for the total sample was 73.94 ± 3.69 (range: 15–75). Detailed demographic and visit characteristics are presented in Table 1.

**Table 1:** Demographic characteristics and overall satisfaction scores (*N* = 1016).

Characteristic	*N* (%)	OSS	Statistical test (*t* or *F*)	*p*	Effect size (*η* ^2^)
Sex			*t* = −1.624	0.105	Cohen's = 0.05
Male	405 (39.86)	74.16 ± 3.01			
Female	611 (60.14)	73.79 ± 4.08			
Age group			*F* = 0.382	0.766	*η* ^2^ < 0.001
≤ 30 years	320 (31.50)	73.97 ± 3.89			
31~40 years	290 (28.54)	74.01 ± 3.10			
41~50 years	188 (18.50)	73.68 ± 5.13			
≥ 51 years	218 (21.46)	74.02 ± 2.47			
Appointment waiting time			*T* = 1.295	0.197	Cohen's = 0.12
On‐time	825 (81.2)	74.04 ± 3.09			
Delayed	191 (18.8)	73.50 ± 5.59			
Total	1016 (100.00)	73.94 ± 3.69			

*Note:* Cohen's *d* and *η* ^2^ represent effect sizes for *t*‐tests and ANOVA, respectively.

Abbreviations: Ns = not significant, OSS = overall satisfaction score.

The mean OSS was 73.94 (SD = 3.70) out of a possible 15–75 range, indicating a high overall satisfaction level. The proportion of participants classified as having high satisfaction (OSS ≥ 60, equivalent to an average item score of ≥ 4 out of 5) was 89.17% (906/1016).

Table 1 presents the comparison of OSS across different demographic and waiting time categories. Independent samples *t*‐tests and one‐way ANOVA revealed no statistically significant differences in OSS based on gender (*t* = −1.624, *p* = 0.105, Cohen's *d* = 0.05), age group (*F* = 0.382, *p* = *0*.766, *η* ^2^ *< *0.001), or waiting time status (*t* = 1.295, *p* = 0.197, Cohen's *d* = 0.12). All effect sizes were small, indicating that demographic characteristics and waiting time were not significant predictors of patient satisfaction in this cohort.

### 3.2 Service‐Quality Dimensions and Correlation With Satisfaction

Table 2 presents the descriptive statistics for the two service‐quality constructs and their bivariate correlations with the OSS.

**Table 2:** One‐stop convenience and communication quality scores and their correlation with overall satisfaction (*N* = 1016).

Construct (items)	Mean (SD)	Range	Pearson's *r* with OSS	95% CI	*p*
One‐stop convenience (11 items)	4.93 (0.25)	1–5	0.992	0.991–0.993	< 0.001
Communication quality (4 items)	4.93 (0.25)	1–5	0.934	0.926–0.942	< 0.001

*Note:* Both constructs showed a pronounced ceiling effect (median = 5.0).

Abbreviations: CI = confidence interval, OSS = overall satisfaction score (range 15–75).

The one‐stop service convenience score (11‐item mean) was 4.93 (SD = 0.25), and the communication quality score (4‐item mean) was also 4.93 (SD = 0.25). The median for both constructs was 5.00, indicating a pronounced ceiling effect, with most patients assigning the maximum rating to nearly all items.

Pearson's correlation analysis revealed strong positive associations between both service‐quality dimensions and OSS. One‐stop convenience showed a near‐perfect positive correlation with OSS (*r* = 0.992, *p* < 0.001, 95% CI 0.991–0.993). Communication quality also exhibited a strong positive correlation with OSS (*r* = 0.934, *p* < 0.001, 95% CI 0.926–0.942). These linear relationships are visually confirmed in Figures 1 and 2, where data points cluster densely in the upper‐right quadrant of both scatter plots, further illustrating the ceiling effect.

**Figure 1:**
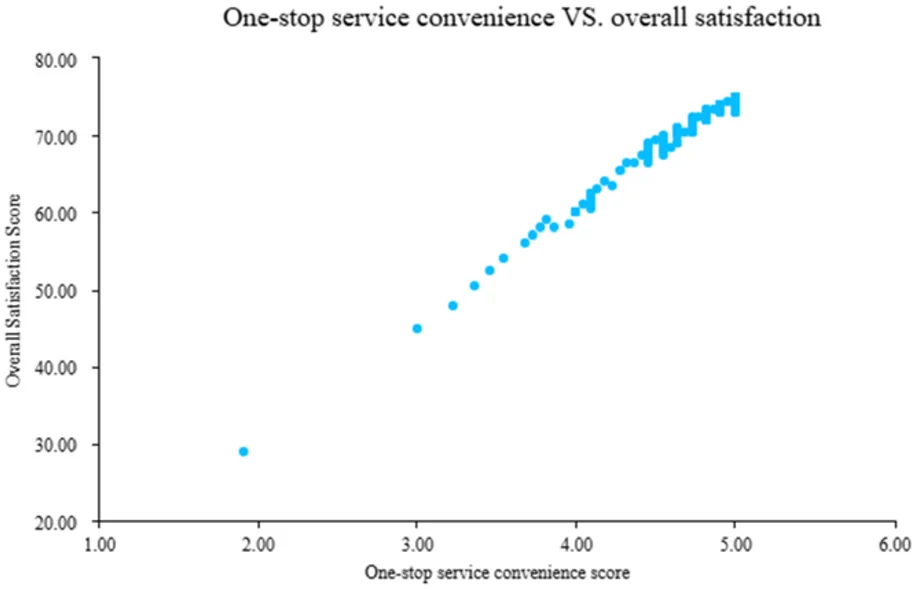
Scatter plot: one‐stop convenience versus overall satisfaction. Scatter plot showing the linear relationship between the one‐stop service convenience score (11‐item mean, range 1–5) and the overall satisfaction score (range 15–75). The solid blue line represents the linear regression fit. The model explained 98.5% of the variance (*R* ^2^ = 0.985, *p *< 0.001). The dense clustering of data points in the upper‐right quadrant illustrates the ceiling effect.

**Figure 2:**
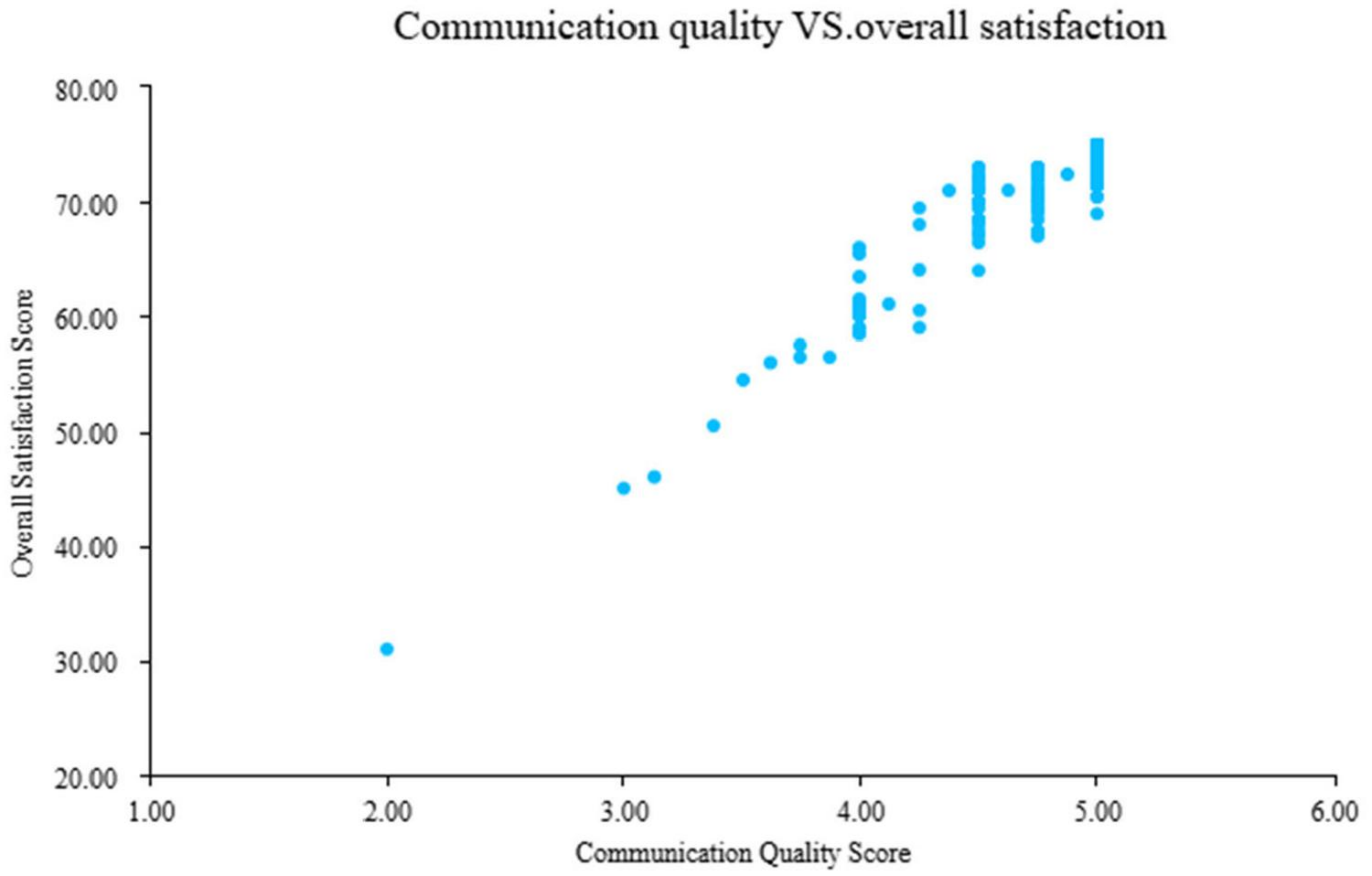
Scatter plot: communication quality versus overall satisfaction. Scatter plot showing the linear relationship between the communication quality score (4‐item mean, range 1–5) and the overall satisfaction score (range 15–75). The solid blue line represents the linear regression fit. The model accounted for 87.3% of the variance (*R* ^2^ = 0.873, *p* < 0.001). A ceiling effect is similarly evident.

Taken together, these findings indicate that both service process efficiency and interpersonal communication quality are strongly associated with patient satisfaction at the bivariate level. However, due to the high intercorrelation between the two constructs (*r* = 0.934), multivariable regression was necessary to disentangle their independent contributions, as presented in Section 3.3.

### 3.3 Hierarchical Regression Analysis

Hierarchical linear regression was performed to identify independent predictors of overall satisfaction (Table 3).

**Table 3:** Hierarchical linear regression predicting overall satisfaction score (*N* = 1016).

Predictor	Model 1 *B* (95% CI)	Model 2 *B* (95% CI)	Model 3 *B* (95% CI)	Standardized *β* (Model 3)	VIF (Model 3)
Constant	74.053** (72.978~75.127)	2.624** (2.062~3.186)	−0.104 (−0.282~0.074)	—	—
Gender (male vs. female)	0.353 (−0.113~0.818)	−0.026 (−0.08~0.032)	0.015 (−0.003~0.032)	0.002	1.012
Age group (Ref: 31–40)	0.012 (−0.192~0.216)	−0.015 (−0.04~0.010)	0.009* (0.002~0.017)	0.003	1.011
Waiting time (delayed vs. on‐time)	−0.536 (−1.11~0.046)	−0.037 (−0.109~0.035)	0.007 (−0.015~0.029)	0.001	1.005
One‐stop convenience (per 1‐point)	—	14.504** (14.393~14.615)	11.206** (11.133~1.278)	0.767	4.733
Communication quality (per 1‐point)	—	—	3.803** (3.729~3.878)	0.254	4.732
Model summary					
*R* ^2^	0.006	0.985	0.999		
Adjusted *R* ^2^	0.003	0.985	0.999		
Δ*R* ^2^	0.006	0.979**	0.014**		
*F*	*F* (3, 1012) = 1.872	*F* (4, 1011) = 16,554.370	*F* (5, 1010) = 146,992.205		
*p*	0.133	< 0.001	< 0.001		

*Note:* Reference categories: female, age 31–40, on‐time appointment. The high *R* ^2^ is interpreted with caution due to the ceiling effect.

Abbreviations: *β* = standardized regression coefficient, *B* = unstandardized regression coefficient, CI = confidence interval, VIF = variance inflation factor.

* *p* < 0.05;

** *p* < 0.01.

Model 1 included only demographic characteristics (gender, age group) and waiting time status. This model explained a negligible proportion of the variance in OSS (*R* ^2^ = 0.006, adjusted *R* ^2^ = 0.003, *F* (3, 1012) = 1.872, *p* = 0.133), and none of the predictors reached statistical significance.

Model 2 added the one‐stop convenience score, which substantially improved the model fit, explaining 98.5% of the variance (*R* ^2^ = 0.985, Δ*R* ^2^ = 0.979, *p* < 0.001). One‐stop convenience was a significant positive predictor (*B* = 14.504, 95% CI 14.393–14.615, *β* = 0.992, *p* < 0.001).

Model 3 added the communication quality score. The full model explained 99.9% of the variance in OSS (*R* ^2^ = 0.999, adjusted *R* ^2^ = 0.999, *F* (5, 1010) = 146, 992.205, *p* < 0.001). Both one‐stop convenience (*B* = 11.206, 95% CI 11.133–11.278, *β* = 0.767, *p* < 0.001) and communication quality (*B* = 3.803, 95% CI 3.729–3.878, *β* = 0.254, *p* < 0.001) remained significant independent predictors. Demographic variables and waiting time status were not significant in the final model (all *p* > 0.05). Collinearity diagnostics indicated acceptable levels (VIF < 5 for all predictors).

A conceptual path model summarizing these findings is presented in Figure 3.

**Figure 3:**
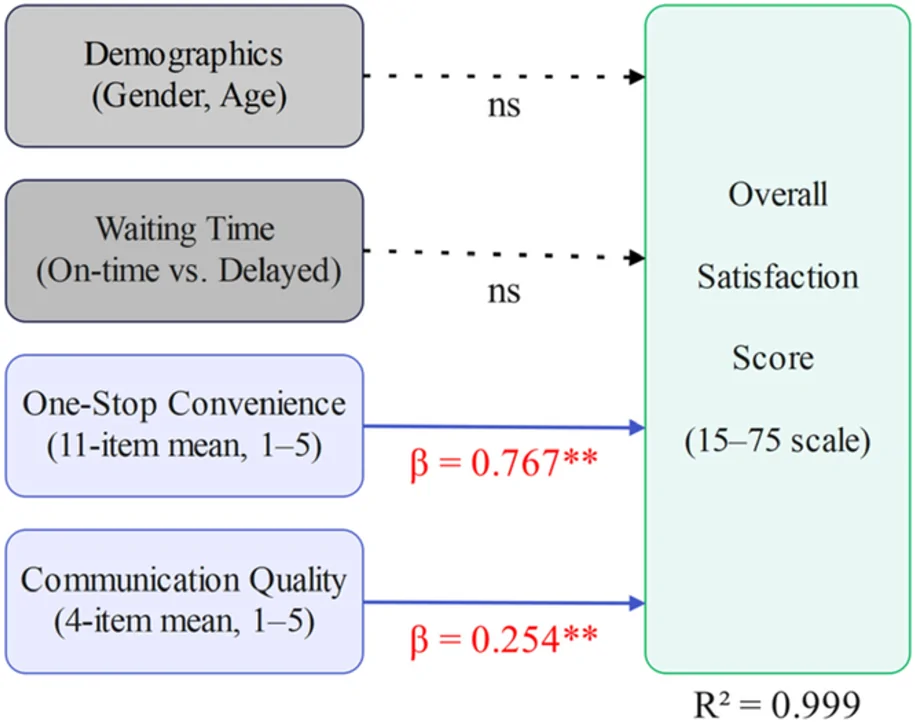
Conceptual path model of predictors of overall satisfaction based on hierarchical regression analysis (*N* = 1016). Solid arrows indicate significant independent predictors in the final model (Model 3, Table 3), with standardized beta coefficients (*β*) shown. Dashed arrows indicate nonsignificant associations (ns, *p* > 0.05). One‐stop convenience (*β* = 0.767, *p* < 0.01) and communication quality (*β* = 0.254, *p* < 0.01) were both significant positive predictors of overall satisfaction. The full model explained 99.9% of the variance (*R* ^2^ = 0.999). **p < 0.01.

### 3.4 Environmental Convenience

Among the 1016 respondents, 962 (94.69%) expressed high recognition of environmental convenience. Within this subgroup, the rate of high satisfaction (OSS ≥ 60) reached 96.67%, further underscoring the importance of the physical environment as a foundational element of patient satisfaction.

## 4 Discussion

### 4.1 Principal Findings and Consistency With Prior Evidence

This study, based on survey data from 1016 outpatients, provides significant evidence for understanding the key factors influencing patient satisfaction in general hospital outpatient departments. The results confirm that one‐stop service convenience (*β* = 0.767, *p* < 0.01) and communication quality (*β* = 0.254, *p* < 0.01) are the two strongest independent predictors of overall satisfaction. The survey revealed a high overall satisfaction level, with patients offering particularly positive evaluations of the integrated services provided by the One‐Stop Service Center, including appointment scheduling, waiting management, guidance, nursing care, and counter services. Furthermore, this study reaffirmed a significant positive correlation between the quality of doctor–patient communication and satisfaction levels. Contrary to some prior findings, this study did not find a significant association between patient age, gender, or waiting time (binary: on‐time vs. delayed) and overall satisfaction in the multivariable model (all *p* > 0.05), though this may be attributable to the ceiling effect observed in the data.

Interestingly, our null finding regarding gender is consistent with Younis et al. [11], who also reported no significant gender difference in satisfaction. However, a notable divergence emerges with respect to age: while Younis et al. [11] found that younger patients reported significantly higher satisfaction, our study did not replicate this age‐related pattern. This discrepancy may stem from differences in sample composition or healthcare context. For instance, the universally high satisfaction scores in our cohort (mean OS = 73.94/75) may have compressed any potential age‐related variation, making it statistically undetectable. Alternatively, the One‐Stop Service Model may be equally effective across all age groups, thereby attenuating age as a differentiating factor. Future studies with more heterogeneous samples are warranted to clarify this relationship.

### 4.2 Theoretical Interpretation Through the SERVQUAL Model

The findings of this study can be effectively interpreted through the SERVQUAL service quality model proposed by Parasuraman et al. [7]. This model posits that service quality stems from the gap between customer expectations and perceptions, measured across five dimensions: reliability, responsiveness, assurance, empathy, and tangibles. In our study, key influencing factors demonstrated a remarkable alignment with this framework: “receiving care within the scheduled appointment time” reflects reliability; “nursing professionalism and response speed” embody responsiveness and assurance; “the quality of doctor communication” encompasses assurance and empathy; and “environmental convenience” directly corresponds to tangibles. Our regression results (Table 3) quantitatively support this framework, showing that the combined tangible (convenience) and intangible (communication) aspects of service explain nearly all the variance in satisfaction (*R* ^2^ = 0.999). This strong alignment indicates that the One‐Stop Service Model, through systematic process re‐engineering, effectively enhances patients' service perceptions across multiple critical dimensions, thereby narrowing the service quality gap.

### 4.3 The Critical Roles of Waiting Time and Environmental Factors

Waiting time has consistently been identified as a key predictor of patient satisfaction [7, 11, 12, 13]. In our study, bivariate analysis revealed a similar trend, though it did not reach statistical significance (*t* = 1.295, *p* = 0.197). Further hierarchical regression analysis (Table 3, Model 1) confirmed that the binary measure of waiting time (on‐time vs. delayed) was not a significant predictor when considered alongside other demographic factors (*R* ^2^ = 0.006, *p* = 0.133). This suggests that in a highly efficient One‐Stop system where the majority of patients (81.2%) were seen on time, the impact of occasional delays may be overshadowed by the overwhelmingly positive perception of the overall service process. The One‐Stop Service Center directly addresses this issue by integrating multiple service windows and optimizing workflows, thereby significantly reducing waiting times, enhancing visit efficiency, and improving the patient experience. Simultaneously, an overwhelming majority of respondents (94.69%, 962/1016) expressed high recognition of the environmental convenience, and within this group, the rate of high satisfaction reached 96.67%. This finding strongly underscores the importance of the Tangibles dimension in the SERVQUAL model—modern facilities, clear signage, and a comfortable, clean waiting environment directly signal professionalism and care to patients, laying the foundation for high satisfaction. Nevertheless, instances of extended waiting still noticeably reduced satisfaction, suggesting a need for further research into the specific impacts of prolonged waiting under different circumstances to develop more targeted improvement strategies.

### 4.4 The Function of One‐Stop Centers in Complex Healthcare Systems

As the primary providers of specialized diagnosis and treatment and bearers of public health tasks, large public tertiary hospitals face the simultaneous challenges of high operational costs, elevated patient expectations, and ever‐increasing outpatient volumes, all of which can negatively impact the patient experience. Consequently, the establishment of One‐Stop Service Centers [14, 15] has emerged as a crucial practice for improving service quality. The findings of this study show that patients generally provided high ratings for guidance services, nursing attitudes, and professional competence. By coordinating processes for registration, payment, pharmacy, and refunds, the One‐Stop Service significantly shortened patient cycle times and enhanced the overall healthcare encounter.

### 4.5 Communication Quality, Perceived Care Quality, and Contextual Adaptation

This study further demonstrates that patient satisfaction is closely linked to perceived medical quality. Specifically, our analysis revealed that communication quality (encompassing physician/nurse attitude and technical competence) was a significant independent predictor of satisfaction (*β* = 0.254, *p* < 0.01), even after controlling for the powerful effect of one‐stop convenience. This finding aligns with research by Waters et al. [8] and Stephens et al. [16], and is supported by Zhang et al. and Donnally et al. [17, 18], all emphasizing the pivotal role of effective communication in enhancing the specialty outpatient experience. Beyond interpersonal communication, the physical environment of the one‐stop service center also plays a contributing role; a clean and comfortable setting has been shown to enhance the overall healthcare experience and patient satisfaction, as highlighted in systematic reviews by Li et al. [19] and empirical work by Redding et al. [1]. When compared to King and colleagues' research in New Zealand [2] and Godley and colleagues' findings in Western contexts [4], this study not only validates the universal value of the “One‐Stop” model across different healthcare systems but, more importantly, reveals its critical adaptation within the Chinese public hospital setting. The key insight is that addressing ultra‐high patient volumes through refined process management—such as the precise control of appointment schedules—is paramount for enhancing satisfaction in this specific context. This underscores the model's flexibility and its ability to be tailored to address unique systemic challenges.

### 4.6 Limitations and Future Research

This study has several limitations. First, its cross‐sectional design identifies associations but not causality. Second, the data were collected from a single tertiary hospital, which may affect the generalizability of findings. Third, a pronounced ceiling effect was observed, with mean scores for both one‐stop convenience (4.93/5.0) and communication quality (4.93/5.0) being extremely high. This restriction of range, while indicative of excellent service, likely inflated the correlation coefficients and may have masked the true independent contribution of these variables relative to each other. Fourth, the use of a binary waiting time variable (on‐time vs. delayed) may have been too coarse to capture the nuanced effects of different delay durations. Future research should employ longitudinal or interventional designs to establish causal relationships and explore the model's effectiveness in diverse healthcare settings across different regions and levels of hospitals. Studies utilizing more granular measures of waiting time (e.g., minutes delayed) and employing analytical techniques less sensitive to ceiling effects (e.g., Tobit regression) are warranted. Qualitative investigations could provide deeper insights into the specific elements of communication and environmental design that are most valued by patients.

### 4.7 Innovation and Contributions

This study offers several novel contributions to the existing literature on patient satisfaction in outpatient settings.

First, while previous studies have often examined service process efficiency and interpersonal communication as separate predictors [9, 12, 16], this study is among the first to simultaneously quantify their relative importance within a unified “One‐Stop” service framework using hierarchical regression. Our results demonstrate that one‐stop convenience (*β* = 0.767) and communication quality (*β* = 0.254) are both significant independent predictors, but with markedly different effect sizes. This finding provides empirical evidence that process efficiency is the dominant driver of satisfaction, yet it does not diminish the distinct and irreplaceable role of humanistic care.

Second, this study extends the application of the SERVQUAL model to the specific context of a Chinese tertiary hospital implementing a centralized service model. While the SERVQUAL framework has been widely validated in Western healthcare settings [11], its explanatory power in a high‐volume, resource‐constrained public hospital environment has been less explored. Our near‐perfect model fit (*R* ^2^ = 0.999) suggests that the tangible (convenience) and intangible (communication) dimensions of SERVQUAL are particularly salient in this context, offering a parsimonious yet powerful explanation for patient satisfaction.

Third, this study provides methodological transparency by explicitly documenting and discussing the ceiling effect. Rather than treating uniformly high scores as a limitation to be glossed over, we interpret them as a meaningful indicator of successful service implementation. This honest appraisal offers a replicable template for future studies facing similar data distribution challenges.

Finally, by identifying that demographic variables (age, gender) and binary waiting time are nonsignificant in the multivariable model, this study challenges the assumption that these factors are universally important predictors. Instead, it suggests that in a well‐designed One‐Stop system, the service model itself may attenuate traditional sources of variation in patient satisfaction.

## 5 Conclusion

This study conclusively demonstrates that the “One‐Stop” service model serves as an effective management strategy for significantly enhancing outpatient satisfaction in large public hospitals. Our findings robustly validate that while a seamlessly integrated service process is the strongest driver of satisfaction, it does not negate the independent and significant importance of high‐quality, empathetic communication from healthcare providers. Continuous optimization of environmental convenience and systematic training in healthcare providers' communication skills are pivotal levers for driving continuous quality improvement in clinical services. By offering a targeted, practical pathway for optimizing service delivery within resource‐constrained environments, this research provides valuable evidence‐based insights for healthcare administrators in China and similar settings aiming to improve patient experience through integrated care models.

## Author Contributions

**Gong Xue:** data curation, project administration, writing – original draft. **Wei Li Li:** conceptualization, formal analysis, resources, data curation. **Chen Xia:** methodology, Software. **Peng Wei Qun:** investigation, supervision, project administration, writing – review and editing. **Ding Xiao Rong:** validation, supervision, writing – review and editing. All authors read and approved the final manuscript.

## Funding

The authors have nothing to report.

## Disclosure

The corresponding author affirms that this manuscript is an honest, accurate, and transparent account of the study being reported; that no important aspects of the study have been omitted; and that any discrepancies from the study as planned have been explained.

## Ethics Statement

As the study relied exclusively on secondary data from publicly available, de‐identified sources, it was exempt from ethical approval. We confirm that all procedures were conducted in accordance with the institutional ethical standards and the principles for medical research involving human subjects as outlined in the Declaration of Helsinki.

## Consent

This cross‐sectional survey, which utilized anonymous questionnaires, qualified for exemption from informed consent.

## Conflicts of Interest

The authors declare no conflicts of interest.

## Supporting information


Supporting File

## Data Availability

The data that support the findings of this study are available from the corresponding authors upon reasonable request. The corresponding authors, Peng Wei Qun and Ding Xiao Rong, had full access to all data in this study and takes complete responsibility for the integrity of the data and the accuracy of the data analysis.
