# Supplementary material for: Patient Satisfaction With a One‐Stop Service Center in a Tertiary Hospital: A Cross‐Sectional Study
Source: Health Sci Rep. 2026 Oct 18;9(10):e73330. doi: 10.1002/hsr2.73330 (PMC13678110; doi:10.1002/hsr2.73330)
Supplement: Supplementary file 1 [file HSR2-9-e73330-s001.docx]

Checklist Title:STROBE (Strengthening the Reporting of Observational Studies in Epidemiology) Checklist for Cross-Sectional Studies

Manuscript Title:**Patient Satisfaction with a One-Stop Service Center in a Tertiary Hospital：A Cross-Sectional Study**

| Section | Item No | Recommendation | Location in  Manuscript | Description of Compliance |
| --- | --- | --- | --- | --- |
| Title & Abstract | 1a | Indicate the study’s design with a commonly used term in the title or abstract. | Title: Page 1, Line 1  Abstract: Page 1, Lines 9-34 | Title includes "Patient Satisfaction with a One-Stop Service Center in a Tertiary Hospital：A Cross-Sectional Study" |
|  | 1b | Provide in the abstract an informative and balanced summary of what was done and what was found. | Page 1, Lines 19-34 | Abstract structured with Results, Conclusion. Key findings (e.g., 89.17% satisfaction) and conclusions are summarized. |
| Introduction | 2 | Explain the scientific background and rationale for the investigation. | Page 2-3, Lines 37-79  Introduction | Background on patient satisfaction, challenges in public hospitals, and the "One-Stop" service model is provided. |
|  | 3 | State specific objectives, including any prespecified hypotheses. | Page 2-3, Lines 71-79  Conclusion | The aim is clearly stated: "...we aimed to⑴..." |
| Methods | 4 | Present key elements of study design early. | Page 3, Lines 82-86 Section 2.1 | The section begins with: "This cross-sectional study was... " |
|  | 5 | Describe the setting, locations, and relevant dates, including periods of recruitment, exposure, follow-up, and data collection. | Page 3, Lines 88-107 Section 2.2-2.3 | Setting: "cl". Dates: "March to April 2025". |
|  | 6a | Give the eligibility criteria, and the sources and methods of selection of participants. | Page 3, Lines 90-93 Section 2.2 | Inclusion criteria are clearly listed: (1) age ≥18; (2) clear consciousness; (3) if patients… Sampling: "convenience sampling method". |
|  | 6b | For matched studies, give matching criteria and the number of exposed and unexposed. |  | **Not Applicable.** This is not a matched study. |
|  | 7 | Clearly define all outcomes, and diagnostic criteria if applicable. | Page 4, Lines 109-122 Section 2.4 | The primary outcome is "Data were collected using…. |
|  | 8 | For each variable of interest, give sources of data and details of methods of assessment. | Page 4-5, Lines 124-156Section 2.5-2.7 | Data source: Data were collected using a self-administered questionnaire |
|  | 9 | Describe any efforts to address potential sources of bias. | Page 4, Lines 124-127 Section 2.5 | States: Content validity was assessed… |
|  | 10 | Explain how the study size was arrived at. | Page 3, Lines 97-107 Section 2.3 | States: he sample size was informed by… |
|  | 11 | Explain how quantitative variables were handled in the analyses. | Page 4-5, Lines 137-156 Section 2.7 | Description of statistical methods: " Data were analyzed using SPSSAU…" |
|  | 12a | Describe all statistical analyses, including methods to control for confounding. | Page 5, Lines 143-154 Section 2.7 | States: "①Pearson correlation analysis…" |
|  | 12b | Explain how missing data were addressed. |  | **Not Specified.** The manuscript does not mention missing data or how it was handled. This can be noted. |
|  | 12c | Describe any sensitivity analyses. |  | **Not Applicable.** No sensitivity analyses were conducted in this univariate analysis. |
| Ethics | 13a | Report approval by an ethics committee and participant consent. | Page 5, Lines 158-162 Section 2.8 | States: "This study used anonymous questionnaires... qualifying for exemption from ethical approval... in compliance with the Declaration of Helsinki." |
|  | 13b | For studies involving animals, report ethical approval and care. |  | **Not Applicable.** This is a human subjects study. |
| Results | 14a | Give numbers of individuals at each stage of the study. | Page 3, Lines 88-95 Section 2.2 | Reports: "A total of 1020 questionnaires were distributed, and 1016 valid questionnaires were collected." |
|  | 14b | Give reasons for non-participation at each stage. |  | **Not Specified.** Reasons for the 4 non-responses are not given. |
|  | 14c | Use a flow diagram if applicable. |  | **Not Applicable.** A flow diagram was not used. |
|  | 15a | Give characteristics of study participants. | Page 5-6, Lines 169-188 Section 3.1 & Table 1 | Demographic Characteristics (gender, age, etc.) are presented in text and Table 1. |
|  | 15b | Indicate the number of participants with missing data for each variable. |  | **Not Specified.** |
|  | 16a | Report unadjusted estimates and confounder-adjusted estimates. | Page 6,  Lines180-188 Section 3.1 | Reports:Table 1 |
|  | 16b | Report category boundaries when continuous variables were categorized. | Page 5-8, Lines 169-252 Section 3.1-3.3 & Table 1-3 | Age groups are clearly defined (e.g., ≤30 years (n=320，31.50%)，31-40 years (n=290，28.54%)"with detailed breakdowns in Table 1. |
|  | 16c | If relevant, consider translating estimates of relative risk into absolute risk. |  | **Not Applicable.** Primarily relative measures (percentages, OR) are reported. |
|  | 17 | Report other analyses done—e.g., subgroups, interactions, sensitivity. |  | **Not Specified.** The analysis focused on univariate associations. |
| Discussion | 18 | Summarize key results with reference to study objectives. | Page8, Lines 260-282  Section 4.1 | The first paragraph summarizes key findings related to the core determinants of satisfaction. |
|  | 19 | Discuss limitations of the study, considering potential biases and imprecision. | Page 11, Lines 348-362  Section 4.6 | Limitations are explicitly discussed: " This study has several limitations.First…" |
|  | 20 | Give a cautious overall interpretation of results and discuss relevance. | Page11-12, Lines 364-391  Sections 4.7 | This study offers several novel contributions… |
|  | 21 | Discuss the generalizability (external validity) of the results. | Page12, Lines 393-403  Section 5.0 | Generalizability is discussed in the context of Chinese public hospitals and as a limitation. |
| **Other** | 22 | Give the source of funding and role of funders. |  | **Not Specified.** If no funding was received, this should be stated in the manuscript. |
